# Enhanced Flexible Piezoelectric Nanogenerators Using Ethanol-Exfoliated g-C_3_N_4_/PVDF Composites via 3D Printing for Self-Powered Applications

**DOI:** 10.3390/nano14191578

**Published:** 2024-09-29

**Authors:** Omkar Y. Pawar, Baoyang Lu, Sooman Lim

**Affiliations:** 1 Graduate School of Flexible and Printable Electronics, LANL-JBNU Engineering Institute-Korea, Jeonbuk National University, Jeonju 54896, Republic of Korea; omkar@jbnu.ac.kr; 2 Jiangxi Province Key Laboratory of Flexible Electronics, Jiangxi Science & Technology Normal University, Nanchang 330013, China

**Keywords:** piezoelectric nanogenerators, g-C_3_N_4_ nanoflakes, ethanol exfoliation, direct ink writing, flexible electronics

## Abstract

This study presents the development of flexible piezoelectric nanogenerators (PENGs) utilizing graphitic carbon nitride (g-C_3_N_4_) nanoflakes (CNNFs) and polyvinylidene fluoride (PVDF) composites fabricated via the direct ink writing (DIW) 3D printing method. A novel approach of synthesizing CNNFs using the ethanol exfoliation method was demonstrated, which significantly reduces preparation time and cost compared to traditional acid exfoliation. The CNNFs are incorporated into PVDFs at varying weight percentages (5, 7.5, 10, and 15 wt.%) to optimize the β-phase content and piezoelectric properties. Characterization techniques including XRD, FTIR, and FESEM confirm the successful synthesis and alignment of nanoflakes inside the PVDF matrix. The film with 7.5% CNNF achieves the highest performance, exhibiting a peak output voltage of approximately 6.5 V under a 45 N force. This study also explores the effects of UV light exposure. Under a UV light, the film exhibits an output voltage of 8 V, indicating the device’s durability and potential for practical applications. The fabricated device showed significant voltage outputs during various human motions, confirming its suitability for wearable self-powered IoT applications. This work highlights the efficacy of the ethanol exfoliation method and the DIW printing technique in enhancing the performance of flexible PENGs.

## 1. Introduction

Self-powered devices have significantly advanced the growth of miniature electronics used for Internet of Things (IoT) applications [1]. These devices have replaced traditional batteries, saved space, and reduced environmental harm by eliminating toxic battery components [2,3,4]. Piezoelectric nanogenerators (PENGs) have emerged as leaders in the field of self-powered devices due to their straightforward fabrication and effective working mechanisms. Various piezoelectric materials, including polymers, single crystals, perovskite, and lead-free perovskite, have been developed to meet specific application requirements [5]. Each material offers unique properties, such as flexibility in polymers [6] and superior performance in perovskites [7]. To enhance the versatility and performance of piezoelectric materials, composite films combining polymers and perovskites have been introduced, enabling the fabrication of flexible, high-performance PENGs [8].

Carbon materials have attracted significant attention due to their biocompatibility, easy synthesis, and availability [9,10]. Multiple carbon-based materials have been introduced to the PENG field in recent years. Recently, graphitic carbon nitride (g-C_3_N_4_) (GCN) has been introduced to the field of PENG. GCN is a well-known candidate for photocatalysis application due to its superior photocatalytic activity [11,12,13,14,15]. GCN is considered as a multifunctional material, utilized in various applications such as photoluminescence [16], photocatalysis [17], energy storage [18], and memory [19]. GCN has also been used for piezoelectric applications, showing piezo-photo properties appreciated in degradation purposes [20]. Having wide band gap of 2.7 eV [21], GCN can function as a visible light-sensitive catalyst. Although it has gained significant attention due to its easy synthesis method, traditional acid-based exfoliation methods are not ideal due to their longer preparation times and potential environmental hazards. Multiple reports been published in recent years, such as Wang et al. [12], who fabricated a piezoelectric nanogenerator based GCN. They synthesized GCN using different precursors and combinations of precursors. They synthesized GCN and recorded the output voltage of PENGs fabricated using GCN synthesized using melamine, urea, or mixture of both precursors. They recorded 1.6 V with an output current of 141 nA/cm^2^ of PENG consisting of GCN synthesized via mixture of precursors. Khalifa et al. [22] fabricated a g-C_3_N_4_-based piezoelectric nanogenerator. In their work, they fabricated a PVDF/GCN piezoelectric film via the electrospinning method. A potential of 20 kV was applied to a syringe needle while fabricating the fibers. The voltage applied during the fabrication process eliminated the need for further pooling processes (poling aligns the dipoles of the material, which improves the output voltage). They achieved an output voltage of 7.5 V using an output current of 0.23 µA. Bayan et al. [11] also fabricated a PVDF/GCN-based PENG via the spin coating method. They achieved a peak output voltage of 2.3 V.

In the present report, we present an ethanol-based exfoliation method for synthesizing GCN nanoflakes (CNNFs), offering a more straightforward, cost-effective, and environmentally sustainable alternative to traditional acid-based methods. The synthesized GCN was composited with polyvinylidene fluoride (PVDF) at varying weight percentages (5, 7.5, 10, and 15 wt.%) to enhance its piezoelectric properties. Composite films were fabricated using the Direct Ink Writing (DIW) method, which facilitates the horizontal alignment of CNNFs inside the PVDF. The performance of the DIW-fabricated films was systematically compared with that of solution-cast films. Additionally, the piezo-photo response of the films was evaluated under UV light exposure. The practical application of the fabricated device was demonstrated by attaching it to different parts of the human body to harvest energy from body movements.

## 2. Material and Method

### 2.1. Materials

Melamine (Sigma Andrich, St. Louis, MO, USA, 99% pure), PVDF (Sigma Aldrich, St. Louis, MO, USA, purity not specified), ethanol (manufacturer and purity not specified), N,N-dimethylformamide (DMF, Sigma Aldrich, 99.8% pure), and sulfuric acid (Sigma Aldrich, 98%) were utilized for the experiments.

### 2.2. Synthesis of GCN Nanoflakes (CNNFs)

CNNFs were synthesized via the polycondensation of melamine at 550 °C in a muffle furnace. A total of 5 g of melamine was weighed and placed in an alumina crucible, then heated in a furnace for 3 h at 550 °C. The temperature was increased at a ramp rate of 5 °C/min until it reached 550 °C. After 3 h of reaction, the furnace was allowed to cool naturally [10]. After cooling, the yellow-colored product was collected and crushed in a mortar and pestle until a fine powder was obtained. The crushed powder was named bulk (stacked nanoflake structure) GCN powder. To synthesize nanoflakes, the powder was then exfoliated via a wet chemical method. We modified the exfoliation by changing the acid exfoliation to ethanol exfoliation, which reduced the preparation time of the CNNFs. First, 1 g of synthesized bulk GCN was added to 70% ethanol and sonicated for 2 h. After sonication, the CNNFs were collected. The CNNF powder was collected and used for further characterization [23]. The ethanol exfoliation procedure provides a simple, cost-effective alternative to acid treatment (Table 1).

### 2.3. Piezoelectric Film Fabrication

The film was prepared via DIW. Different weight percentages of CNNFs were added to 10 mL of DMF and sonicated for 1 h. After obtaining a uniformly dispersed CNNF solution, 1 g of PVDF was added, and the solution was stirred overnight at 60 °C. After the overnight stirring, the solution was transferred to a DIW extruder and printed on an ITO-coated PET substrate with fixed parameters. The same procedure was repeated for all concentrations of CNNF (5, 7.5, 10, 15 wt.%). After printing, the film was dried using a vacuum oven. A yellow, square-shaped film measuring 2 × 2 cm² was obtained after the drying process. The film was flexible and strong.

### 2.4. Characterizations

Field emission scanning electron microscopy (FESEM) was performed to analyze the morphology and horizontal alignment of nanostructures in a printed film at 1–5 kV, conducted at the Center of the University-Wide Research Facility (CURF) at Jeonbuk National University. X-ray diffraction (XRD) utilizing a Cu Kα source (wavelength: 0.154 nm) using a D8 Advance diffractometer manufactured by Bruker, Germany, was used to characterize the synthesized material. Fourier-transform infrared (FTIR) spectroscopy using a Bomen MB 100 instrument was utilized to identify the crystalline phases and interfacial interactions within the printed films. The piezoelectric properties of the printed films were evaluated using a quasistatic d33 meter (YE2730A, Sinocera Yangzhou, China), while the dielectric constants and loss tangents of the as-printed samples were measured across frequencies ranging from 1 kHz to 1 MHz using an LCR meter (IM3570, Hioki Nagano, Japan). A digital oscilloscope (KEYSIGHT DSOX2012A) (Swindo, UK) was used to analyze the piezoelectric performance, and an electrometer (Keithley 2450, Solon, OH, USA) measured the voltage and current produced by the printed film sandwiched between nickel tape, with a pressure sensor employed to record the applied force during these measurements.

## 3. Result and Discussion

**Figure 1:**
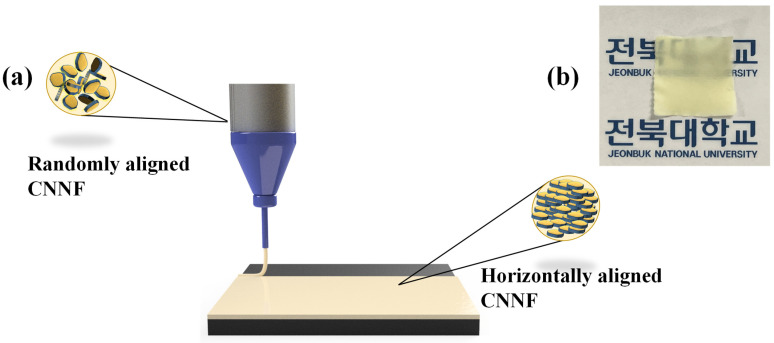
(**a**) Illustration of PVDF/CNNF film fabrication using the DIW method. (**b**) Image of fabricated film after drying.

Figure 1a illustrates the printing of PVDF/CNNF piezoelectric film onto an ITO-coated PET substrate using the DIW method. First, we introduced the prepared ink into the DIW extruder for printing. When the printing program was executed, pressure was applied to the ink, causing it to flow from the needle, where it experienced a high pressure. With excess pressure, the viscosity of the ink at the needle was drastically reduced. This condition facilitated the alignment of the CNNFs present in the ink in the horizontal direction. The horizontal alignment significantly contributed to improving the performance of the fabricated film. After successful printing, the film was transferred to a vacuum oven for drying. The solvent present after printing evaporated during the drying process. After drying, the flakes were permanently aligned in a horizontal direction. Figure 1b shows an image of the fabricated film after the drying process. The fabricated film was yellow in color. Further characterizations such as FTIR, FESEM, etc. were performed for a better understanding of the film’s chemical interactions and the alignment of the nanoflakes in the PVDF matrix. The details of the film will be explained in a later section.

**Figure 2:**
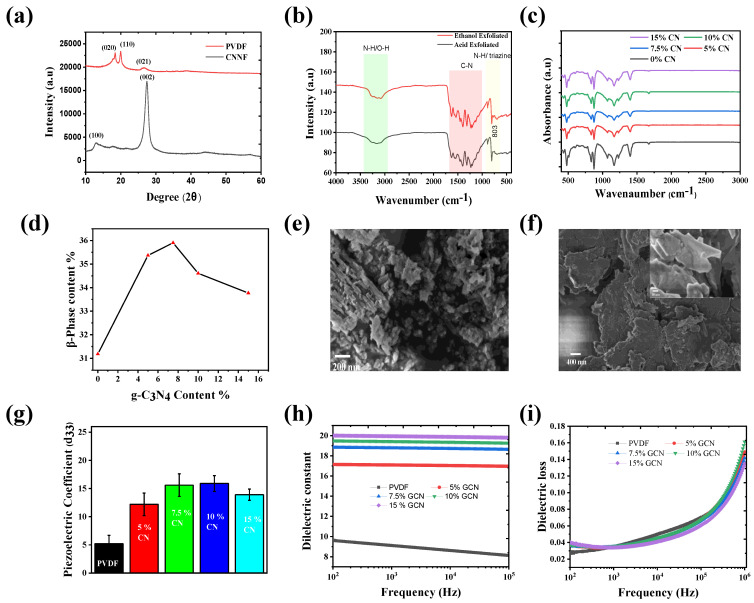
(**a**) XRD of GCN powder and pure PVDF. (**b**) FTIR pattern of CNNF (acid-exfoliated and ethanol-exfoliated). (**c**) FTIR pattern of PVDF/CNNF with different concentrations of CNNF (0, 5, 7.5, 10, and 15 wt.%). (**d**) β-phase of PVDF/CNNF. (**e**) FESEM image of bulk GCN. (**f**) FESEM image of PVDF/CNNF film; inset, magnified FESEM image of fabricated film (PVDF/CNNF). (**g**) d_33_ value of PCDF/CNNF film with GCN 0, 5, 7.5, 10, and 15%. (**h**) dielectric constant of PVDF/CNNF (GCN 0,5, 7.5, 10 and 15%). (**i**) Dielectric loss.

Figure 2a displays the XRD pattern of the as-prepared material. The XRD pattern confirms the successful synthesis of CNNF. Two major peaks are seen in the XRD spectra at 13.1° and 27.4°, corresponding to the (100) and (002) reflections, respectively. The peak present at 13.1°, indexed as (100), represents the plane of graphitic material, while the peak present at 27.4°, indexed as (002), indicates interplanar stacking of the conjugated atomic system. Note that the XRD lines are weaker than those of the bulk GCN, which may be due to the exfoliation process [24]. The XRD of pure PVDF is also provided in Figure 2a. Three peaks are observed in the XRD pattern of PVDF, indexed as (020), (110), and (021) at 18.2°, 20.2°, and 26.5°, respectively. The peaks indexed at (020) and (021) belong to the α-phase, while the peak indexed at (110) belongs to the β-phase.

Figure 2b shows the FTIR pattern of the synthesized CNNF (acid-exfoliated and ethanol-exfoliated), providing information in the form of different peaks. The peak present at around 3000–3500 cm⁻¹ confirms the presence of an N-H group. The peaks located between 1570 and 1634 cm⁻¹ provide evidence of C=N, while the presence of C-C was confirmed by peaks in the range of 1258–1480 cm⁻¹. The peak at 810 cm⁻¹ corresponds to CN tri-s-triazine/triazine units. In the acid-exfoliated CNNFs, the dominant functional group observed was the hydroxyl group (-OH), appearing around 3000–3400 cm⁻¹ in the FTIR spectrum. This formation likely results from the addition of water after exfoliation in acidic media, which facilitates the creation of -OH groups. Additionally, a carboxyl group (-COOH) was detected at around 1700 cm⁻¹. The presence of -COOH groups is common when GCN interacts with acids, as these groups form through acid reactions with surface functionality [25]. In contrast, the ethanol-exfoliated CNNFs showed a broad peak at 3400 cm⁻¹, corresponding to hydroxyl stretching. All the other peaks were seen in the ethanol-exfoliated CNNFs. The higher peak intensity in the ethanol-exfoliated CNNFs compared to the acid-exfoliated samples may be attributed to less structural damage incurred during the gentler exfoliation process. The gentle exfoliation process helps preserve peak intensity [17]. A FTIR investigation was performed on the PVDF/CNNF composite films, providing crucial information about the increase in β-phase with increasing CNNF concentrations. In Figure 2c, we compare the FT-IR patterns of films with different concentrations of CNNFs (0%, 5%, 7.5%, 10%, and 15% wt.%). With a 7.5 wt.% concentration of CNNFs, the β-phase was seen to increase. The bands at 770 cm⁻¹ and 835 cm⁻¹ were considered as identifier bands of the β-phase [10]:

$$F\beta =\frac{A\beta }{1.3\left(A\alpha +A\beta \right)}$$

where Aβ and Aα represent the β-phase and α-phase concentrations, respectively. The piezo-active β-phase increased with increasing contents of CNNFs in the PVDF matrix. The highest β-phase content of 35.60% was observed when the CNNF concentration was 7.5 wt.% of PVDF, after which the performance decreased at CNNF concentrations of 10 and 15 wt.%. The increasing β-phase is considered to be the result of dipole–dipole interactions between CNNF and PVDF, while the decrease in the β-phase at 10 and 15 wt.% may be due to aggregation during the printing procedure. The aggregation of CNNF hinders the dispersion and alignment of nanoflakes in PVDF, leading to a reduction in β-phase content. Apart from the β-phase, FTIR provides information about molecular interactions. The bonds present at around 600, 771, and 949 cm⁻¹ represent the α-phase. The bands at 600, 771, and 949 cm⁻¹ are assigned to the bending vibration of CF₂, whereas the band at 835 cm⁻¹ is attributed to the CH₂ rocking vibration. Asymmetric stretching of CH₂ bonds is present at 2981 cm⁻¹, while symmetric stretching is observed at 3023 cm⁻¹ [26,27,28]. To confirm successful exfoliation of the CNNFs, FE-SEM images before and after exfoliation were compared, leading to the conclusion that CNNFs were successfully formed. Figure 2f shows the alignment of CNNFs incorporated in PVDF, investigating the orientation of the fabricated piezoelectric film. The β-phase of the fabricated film was also investigated using XRD (Supplementary Figure S3). As shown in the FE-SEM image, the CNNFs are oriented in the horizontal plane (Figure 2f). EDS mapping was performed to investigate the effects of exfoliation on the material (Supplementary Figure S2). Additionally, a map sum spectrum is provided (Supplementary Table S1). Due to the shear force experienced during printing, all the nanoflakes were aligned in the same plane. TEM characterization was performed for the synthesized materials (bulk GCN, acid-exfoliated samples, and ethanol-exfoliated samples) (Supplementary Figure S1).

Figure 2g provides information about the d₃₃ value of the fabricated films. PVDF with 0% CNNF exhibits a d₃₃ value of 5.2 pC/N, whereas when the CNNF concentration increases to 7.5%, the d₃₃ value increases to 15.9 pC/N, then decreases as the concentration of CNNF further increases to 15%. Figure 2h presents the dielectric properties of the printed films with different concentrations. As the CNNF concentration increased, the dielectric constant value also increased. The high filler concentration elevated interfacial polarization between the CNNFs filler and PVDF. However, higher concentrations of material also caused agglomeration of the nanoflakes in the PVDF. The effects of agglomeration were reflected in the d₃₃ values (Figure 2h), where the film with a higher dielectric constant (15% CNNF) showed a lower performance. This lower performance of the 15% CNNF film may have been due to nanoflake aggregation, which disrupts stress transfer and alignment, negatively affecting piezoelectric performance. The dielectric loss shown in Figure 2i remained within the accepted range for all concentrations [29,30].

### Piezoelectric Performance

**Figure 3:**
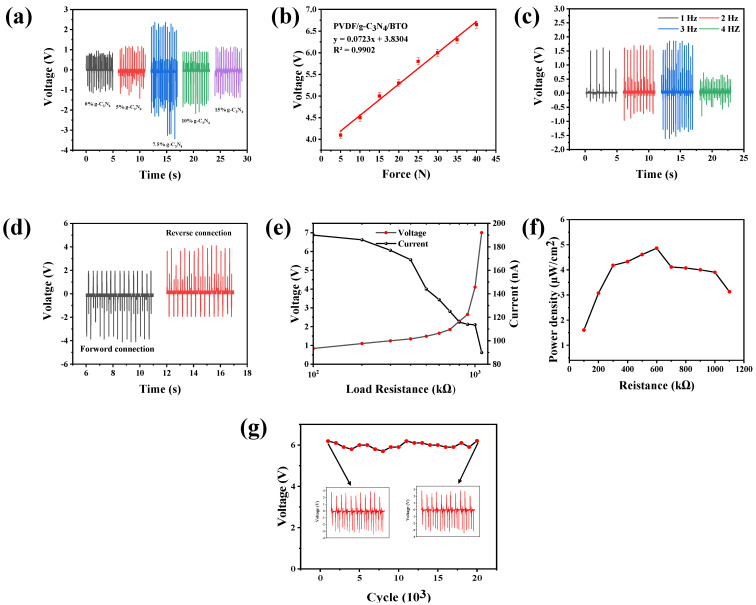
(**a**) Output voltage of the PVDF/GCN film (GCN 0, 5, 7.5, 10, and 15 wt.%). (**b**) Voltage produced by the PVDF/GCN 7.5% film with an applied force in Newtons. (**c**) Frequency output voltage of PVDF/GCN 7.5%. (**d**) Forward and reverse bias voltage of PVDF/GCN 7.5%. (**e**) Voltage and current values of PVDF/GCN across a resistance of 10. (**f**) Power density of PVDF/GCN 7.5 wt.% film. (**g**) Cyclic stability test.

The performance of the fabricated films with different concentrations of CNNFs (0, 5, 7.5, 10, 15 wt.% of PVDF) was investigated, among which the film with 7.5 wt.% concentration of CNNFs showed a higher β-phase value. Therefore, further piezoelectric characterization was carried out, where the film with a CNNF concentration of 7.5 wt.% provided higher performance compared to the other films shown in Figure 3a. As the film with a 7.5 wt.% concentration of CNNFs showed better performance, further performance characteristics were investigated for the same film. Piezoelectric performance was investigated via different tests such as sensitivity, detection of available frequency, and power density. The load sensitivity of the film was investigated by recording the voltage of the fabricated device under an applied force (5–40 N) (Figure 3b) with a constant frequency of 3 Hz. the output voltage values linearly increased as the applied force increased; at 40 N, the peak voltage was recorded ≈ 6.5 V. The fabricated film with CNNF 7.5 wt.% was tested under different frequencies (1, 2, 3, and 4 Hz). The output voltage increased with increasing frequency (1.9 V to 3.4 V), and the voltage decreased at 4 Hz. This decrease in the voltage was due to the escape of charge and accumulation of charge on the surface [31]. To confirm that the generated voltage was due to the piezoelectric effect, the devices were tested under forward and reverse connections. A positive signal was recorded when the device formed a forward connection, and a negative signal was recorded when the device formed a reverse connection. This investigation proved that the output performance was generated due to the piezoelectric effect shown in Figure 3d.

To determine the actual potential of the device in practical applications, external resistance was connected across the device, and the voltage and current values were recorded. As expected, the voltage of the device was seen to increase as the external load resistance increased, while the output current decreased with increasing load resistance. The power density was calculated based on the obtained values using the formula P = I²R/A, where I is the current generated by the fabricated device, R is the load resistance connected, and A is the active area. The peak power density was achieved at 0.6 MΩ, which was 4.86 µW/cm², as shown in Figure 3f.

The printing method chosen for film fabrication plays an important role by facilitating CNNF alignment, resulting in all nanoflakes being aligned along the x-axis, which improves stress transfer and ultimately enhances the output of the device. Lastly, a long cyclic stability test was performed, with constant tapping at a force of 40 N and a frequency of 3 Hz. Throughout the test, the device retained an output voltage of 6.2 V for up to 5000 cycles. Table 2 provides a comparison of previous work with the present study.

## 4. Impact of Printing Parameters

**Figure 4:**
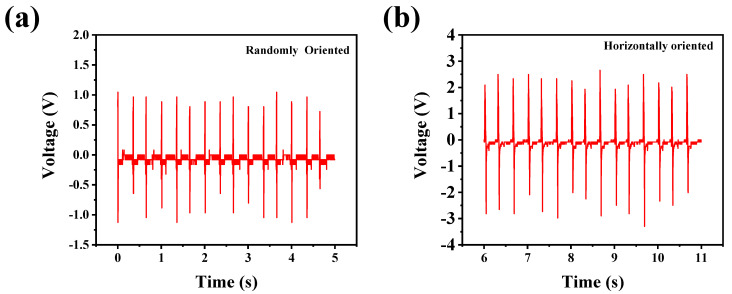
(**a**) Output performance of film fabricated via solution casting. (**b**) Output performance of film fabricated via DIW.

In this work, we also investigated the impact of the printing parameters on device performance. Printing parameters such as printing pressure were the major components for CNNF alignment. To better understand the role of printing pressure, we fabricated films using different methods. Films with optimized CNNF concentrations were fabricated via both solution casting and DIW using the same printing parameters. The size of both films was maintained at 2 × 2 cm². The films were tested under a 40 N force at 3 Hz, with the printed film providing a better performance than the film fabricated via the solution casting method. The obtained results provide valuable information about the importance of the printing method. Without external pressure, it is very difficult for the nanostructures to align on their own. Consequently, the CNNFs were randomly oriented in the solution-cast film, resulting in less stress transfer and lower output voltage compared to the DIW-printed film. Figure 4b shows the output voltage of the printed film, which is 6 V; double the voltage generated by the solution-cast film, which produced approximately 2.6 V under a 40 N force at 3 Hz, as shown in Figure 4a.

## 5. Effects of UV Light

**Figure 5:**
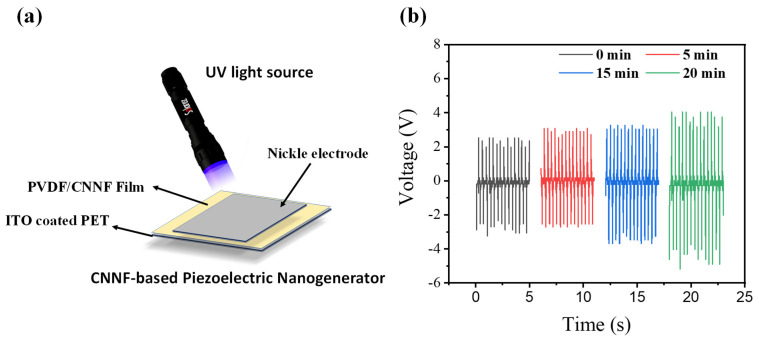
(**a**) Schematic diagram of fabricated device irradiated with UV light. (**b**) Voltage recorded after different time intervals.

The device was tested under UV light to investigate the applicability of the material in harsh environments. Additionally, UV light has the ability to affect the β-phase of PVDF [32,33]. We set up an experiment where a UV source was adjusted to directly illuminate the fabricated film (Figure 5a). The ITO-coated PET face was kept facing upward, allowing for maximum light incidence on the surface of the PVDF/CNNF film. To investigate the effects of UV light, the device was kept under the UV light, with damping applied with a 40 N force and 3 Hz frequency. The output voltage was recorded every 5 min for up to 20 min to observe considerable changes in performance. The performance was seen to increase from 6 V to 8 V when the device was kept under the UV light and subjected to damping with a force of 40 N at 3 Hz. We observed that a considerable change in performance occurred after 15 min, after which there was no significant change. After keeping the device under the same conditions for 20 min, it generated an output voltage of approximately 8 V, representing a considerable performance increase within 20 min shown in Figure 5b.

The increase in the output performance of the fabricated device was likely due to the reduction in the screening effect produced by the CNNF, which is responsible for increased charge/electron carriers [32]. This investigation led us to conclude that the fabricated device can successfully function under harsh conditions, including UV exposure.

## 6. Application

**Figure 6:**
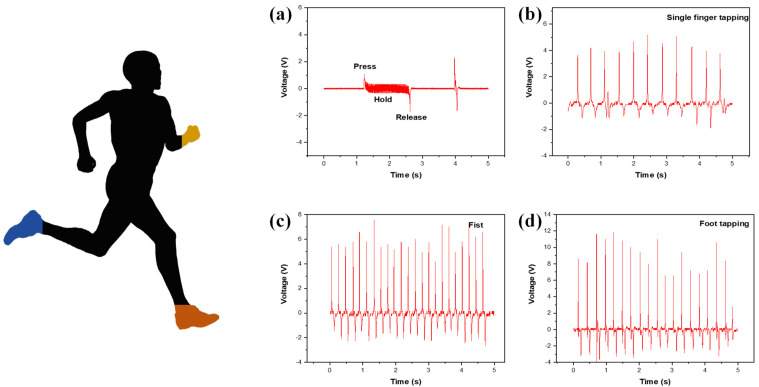
The fabricated device is attached to different parts of the human body. (**a**) Output voltage produced via bending pressing movement. (**b**) Output voltage generated via single finger tapping. (**c**) Voltage generated via fist strike. (**d**) Voltage generated via foot tapping.

Figure 6 shows the output voltage versus time for practical applications. The fabricated device was tested on day-to-day human body kinematics. The device was subjected to bending and stretching, which provided an output voltage of 3.9 V (Figure 6a). After the bending and pressing movement, the voltage was recorded under normal finger tapping. The voltage generated using a single finger is shown in Figure 6b. The interaction of a single finger with the device led to a yield of 6.7 V, as shown in Figure 6b. The device was then attached to the fist, and the recorded voltage provided was 10.1 V (Figure 6c). Finally, the device was integrated with footwear, and a stepping movement was performed. This stepping movement provided a high output voltage of 15.4 V (Figure 6d). This series of tests led us to conclude that the fabricated device is suitable for wearable applications. These tests also provide evidence of the device’s flexibility and durability.

## 7. Conclusions

In this work, we successfully synthesized CNNF via a cost-effective method and fabricated a PVDF/CNNF flexible PENG using the DIW printing method. The modification in the exfoliation procedure reduced the production time and cost of the synthesis process. In addition to the exfoliation modification, multiple strategies were applied to improve the piezoelectric performance of the fabricated device. First, different concentrations (5, 7.5, 10, 15 wt.%) of CNNFs were added into the PVDF, and the β-phase content was improved. The DIW technique was utilized to enhance the alignment of the nanostructures in the PVDF matrix, which further improved the performance of the fabricated device. The effectiveness of the printing technique was confirmed by comparing the performance of the printed film with a solution-cast film, where the printed film provided better performance. Finally, UV light was used to reduce the screening effects on the material, improving its performance potential. By combining these strategies, we achieved a peak voltage of 8 V. The UV test revealed a new approach to improve the performance of the fabricated device and provided evidence of its durability under harsh lighting conditions.

## Figures and Tables

**Table 1:** Comparison of acid-based and ethanol-based exfoliation methods for GCN synthesis.

	Acid Exfoliation	Ethanol Exfoliation
**Solution**	Sulfuric acid (H_2_SO_4_)	Ethanol
**Procedure time**	8 h	1 h
**Post-procedure**	1 h sonication + 1 h of heat treatment at 200 °C	Simple filtration + dry at 60 °C for 1 h
**Cost**	Higher	Lower
**Environmental impact**	Higher (due to acid use)	Lower

**Table 2:** Information about the exfoliation agent used in previous reports and their piezoelectric properties exhibited.

Material	Fabrication	Sample Dimension	Poling	Exfoliation	β-Phase%	Voltage	REF
**g-C_3_N_4_**	Solution casting	3 × 3 cm^2^	no	No	Not provided	1.6 V	[12]
**PVDF/CNNS**	Spin coating	3.0 × 2.0 cm^2^ × 1 mm	no		Not provided	10.2 V With 50 N 2.3 V Finger tapping	[11]
**PVDF/CNNS**	Electrospinning	Not provided	20 kV	Not provided	82 %	~7.5 V	[22]
**PVDF/CNNS/PANI**	Electrospinning	3 × 3 cm^2^ × 0.05 mm	18 kV	HCL	96.9 %	∼30 V	[13]
**PVDF/CNNS**	Direct ink writing	2 × 2 cm^2^ × 0.083 mm	no	H_2_SO_4_	35.90 %	6.5 with 40 N	Previous work
**PVDF/CNNF**	Direct ink writing	2 × 2 cm^2^ × 0.083 mm	no	Ethanol exfoliation	34 %	6.7 V with 40 N	This work

## Data Availability

The data presented in this study are available on request from the corresponding author.

## Supplementary Materials

The following supporting information can be downloaded at: https://www.mdpi.com/article/10.3390/nano14191578/s1. Figure S1. (a–c) TEM image of Bulk, Acid exfoliated and ethanol exfoliated CNNFs. Figure S2. (a) EDS mapping of acid exfoliated CNNF, (b) EDS mapping of ethanol exfoliated. Table S1. Map sum spectrum of acid exfoliated and ethanol exfoliated CNNF. Figure S3. XRD of pure PVDF and film prepared with different concentration of CNNF.

## References

[1] You C.Y., Hu B.F., Xu B.R., Zhang Z.Y., Wu B.M., Huang G.S., Song E.-M., Mei Y.-F. (2022). Foldable-circuit-enabled miniaturized multifunctional sensor for smart digital dust. Chip.

[2] Chen Y., Kang Y., Zhao Y., Wang L., Liu J., Li Y., Liang Z., He X., Li X., Tavajohi N. (2021). A review of lithium-ion battery safety concerns: The issues, strategies, and testing standards. J. Energy Chem..

[3] Giordano M., Mayer P., Magno M. (2020). A Battery-Free Long-Range Wireless Smart Camera for Face Detection. Proceedings of the 8th International Workshop on Energy Harvesting and Energy-Neutral Sensing Systems.

[4] Chen W., Liang J., Yang Z., Li G. (2019). A review of lithium-ion battery for electric vehicle applications and beyond. Energy Procedia.

[5] Pawar O.Y., Patil S.L., Redekar R.S., Patil S.B., Lim S., Tarwal N.L. (2023). Strategic Development of Piezoelectric Nanogenerator and Biomedical Applications. Appl. Sci..

[6] Bouhamed A., Binyu Q., Böhm B., Jöhrmann N., Behme N., Goedel W.A., Wunderle B., Hellwig O., Kanoun O. (2021). A hybrid piezoelectric composite flexible film based on PVDF-HFP for boosting power generation. Compos. Sci. Technol..

[7] Bagheri M.H., Khan A.A., Shahzadi S., Rana M.M., Hasan M.S., Ban D. (2024). Advancements and challenges in molecular/hybrid perovskites for piezoelectric nanogenerator application: A comprehensive review. Nano Energy.

[8] Bairagi S., Ali S.W. (2020). Investigating the role of carbon nanotubes (CNTs) in the piezoelectric performance of a PVDF/KNN-based electrospun nanogenerator. Soft Matter.

[9] Bai X., Wang L., Zong R., Zhu Y. (2013). Photocatalytic activity enhanced via g-C_3_N_4_ nanoplates to nanorods. J. Phys. Chem. C.

[10] Patil S.L., Redekar R.S., Pawar O.Y., Kundale S.S., Sutar S.S., More K.V., Chavan V.D., Kim D.-K., Dongale T.D., Tarwal N.L. (2023). Precursor-dependent resistive switching properties of nanostructured g-C3N4: Statistical and experimental investigations. J. Mater. Sci. Mater. Electron..

[11] Bayan S., Bhattacharya D., Mitra R.K., Ray S.K., Ray S.K. (2020). Self-powered flexible photodetectors based on Ag nanoparticle-loaded g-C3N4nanosheets and PVDF hybrids: Role of plasmonic and piezoelectric effects. Nanotechnology.

[12] Wang R.C., Lin Y.C., Chen H.C., Lin W.Y. (2021). Energy harvesting from g-C_3_N_4_ piezoelectric nanogenerators. Nano Energy.

[13] Khalifa M., Anandhan S. (2019). PVDF Nanofibers with Embedded Polyaniline-Graphitic Carbon Nitride Nanosheet Composites for Piezoelectric Energy Conversion. ACS Appl. Nano Mater..

[14] Li N., Gao X., Su J., Gao Y., Ge L. (2023). Metallic WO2-decorated g-C_3_N_4_ nanosheets as noble-metal-free photocatalysts for efficient photocatalysis. Chin. J. Catal..

[15] Patnaik S., Behera A., Parida K. (2021). A review on g-C3N4/graphene nanocomposites: Multifunctional roles of graphene in the nanohybrid photocatalyst toward photocatalytic applications. Catal. Sci. Technol..

[16] Zhang YPan Q., Chai G., Liang M., Dong G., Zhang Q., Qiu J. (2013). Synthesis and luminescence mechanism of multicolor-emitting g-C_3_N_4_ nanopowders by low temperature thermal condensation of melamine. Sci. Rep..

[17] Nguyen P.A., Nguyen T.K.A., Dao D.Q., Shin E.W. (2022). Ethanol Solvothermal Treatment on Graphitic Carbon Nitride Materials for Enhancing Photocatalytic Hydrogen Evolution Performance. Nanomaterials.

[18] Khasim S., Pasha A., Lakshmi M., Panneerselvam C., Ullah M.F., Darwish A.A.A., Hamdalla T.A., Alfadhli S., Al-Ghamdi S.A. (2022). Synthesis of g-C_3_N_4_/CuO Nanocomposite as a Supercapacitor with Improved Electrochemical Performance for Energy Storage applications. Int. J. Electrochem. Sci..

[19] Patil S.L., Pawar O.Y., Patil H.S., Sutar S.S., Kamble G.U., Kim D.K., Kim J.H., Kim T.G., Kamat R.K., Dongale T.D. (2023). The g-C_3_N_4_-TiO_2_ nanocomposite for non-volatile memory and artificial synaptic device applications. J. Alloys Compd..

[20] Bera B., Das Sarkar M. (2016). Piezoelectric Effect, Piezotronics and Piezophototronics: A Review. Imp. J. Interdiscip. Res. (IJIR).

[21] Yu Y., Liu K., Zhang Y., Xing X., Li H. (2022). High Photocatalytic Activity of g-C_3_N_4_/La-N-TiO_2_ Composite with Nanoscale Heterojunctions for Degradation of Ciprofloxacin. Int. J. Environ. Res. Public Health.

[22] Khalifa M., Mahendran A., Anandhan S. (2019). Synergism of graphitic-carbon nitride and electrospinning on the physico-chemical characteristics and piezoelectric properties of flexible poly(vinylidene fluoride) based nanogenerator. J. Polym. Res..

[23] Iqbal N., Afzal A., Khan I., Khan M.S., Qurashi A. (2021). Molybdenum impregnated g-C_3_N_4_ nanotubes as potentially active photocatalyst for renewable energy applications. Sci. Rep..

[24] Liu Y., Shen S., Li Z., Ma D., Xu G., Fang B. (2021). Mesoporous g-C_3_N_4_ nanosheets with improved photocatalytic performance for hydrogen evolution. Mater. Charact..

[25] Pattnaik S.P., Behera A., Martha S., Acharya R., Parida K. (2019). Facile synthesis of exfoliated graphitic carbon nitride for photocatalytic degradation of ciprofloxacin under solar irradiation. J. Mater. Sci..

[26] Cai X., Lei T., Sun D., Lin L. (2017). A critical analysis of the α, β and γ phases in poly(vinylidene fluoride) using FTIR. RSC Adv..

[27] Salimi A., Yousefi A.A. (2003). FTIR studies of β-phase crystal formation in stretched PVDF films. Polym. Test.

[28] Lanceros-Méndez S., Mano J.F., Costa A.M., Schmidt V.H. (2001). FTIR and DSC studies of mechanically deformed β-pvdf films. J. Macromol. Sci. Part B.

[29] Zhou L., Yang T., Fang Z., Zhou J., Zheng Y., Guo C., Zhu L., Wang E., Hou X., Chou K.C. (2022). Boosting of water splitting using the chemical energy simultaneously harvested from light, kinetic energy and electrical energy using N doped 4H-SiC nanohole arrays. Nano Energy.

[30] Xue Y., Yang T., Zheng Y., Wang K., Wang E., Wang H., Zhu L., Du Z., Wang H., Chou K.-C. (2023). Heterojunction Engineering Enhanced Self-Polarization of PVDF/CsPbBr_3_/Ti_3_C_2_Tx Composite Fiber for Ultra-High Voltage Piezoelectric Nanogenerator. Adv. Sci..

[31] Li H., Lee H.B., Kang J.W., Lim S. (2023). Three-dimensional polymer-nanoparticle-liquid ternary composite for ultrahigh augmentation of piezoelectric nanogenerators. Nano Energy.

[32] Sharma C., Gupta M.K., Badatya S., Srivastava A.K., Sathish N. (2023). Blue light emitting piezoelectric few-layered borophene nanosheets for flexible nanogenerators. Commun. Mater..

[33] Dashtizad S., Alizadeh P., Yourdkhani A. (2021). Improving piezoelectric properties of PVDF fibers by compositing with BaTiO_3_-Ag particles prepared by sol-gel method and photochemical reaction. J. Alloys Compd..

