# Supplementary material for: Enhanced Flexible Piezoelectric Nanogenerators Using Ethanol-Exfoliated g-C3N4/PVDF Composites via 3D Printing for Self-Powered Applications
Source: Nanomaterials (Basel). 2024 Sep 29;14(19):1578. doi: 10.3390/nano14191578 (PMC11478031; doi:10.3390/nano14191578)
Supplement: Supplementary file 1 [file nanomaterials-14-01578-s001.zip › nanomaterials-3144862-supplementary.pdf]

Supporting information

Enhanced Flexible Piezoelectric Nanogenerators Using Ethanol-Exfoliated g-C_3N_4/PVDF Composites via 3D Printing for Self-Powered Applications

TEM of synthesized material

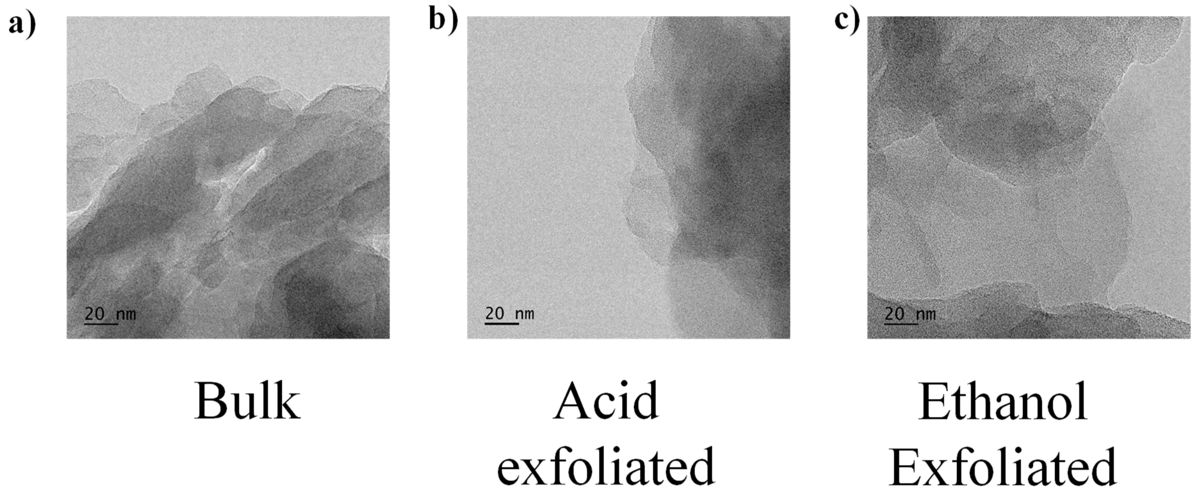

Figure S1. (a–c) TEM image of Bulk, Acid exfoliated and ethanol exfoliated CNNFs.

Figure 1 a represents the TEM image of bulk GCN as the name suggest the bulky structure was seen which represents the typical structure of stacked GCN. The figure 1b provide the slightly less bulky structure which was due to the Acid exfoliation. The figure 1c was image of ethanol exfoliated CNNF we can see the separate flacks.

Both the flacks TEM image almost looks same the difference was noticed in surface functionalization, due to acid harsh exfoliation more oxygen was detected on the CNNF while the EDS characterization than the ethanol exfoliated CNNF

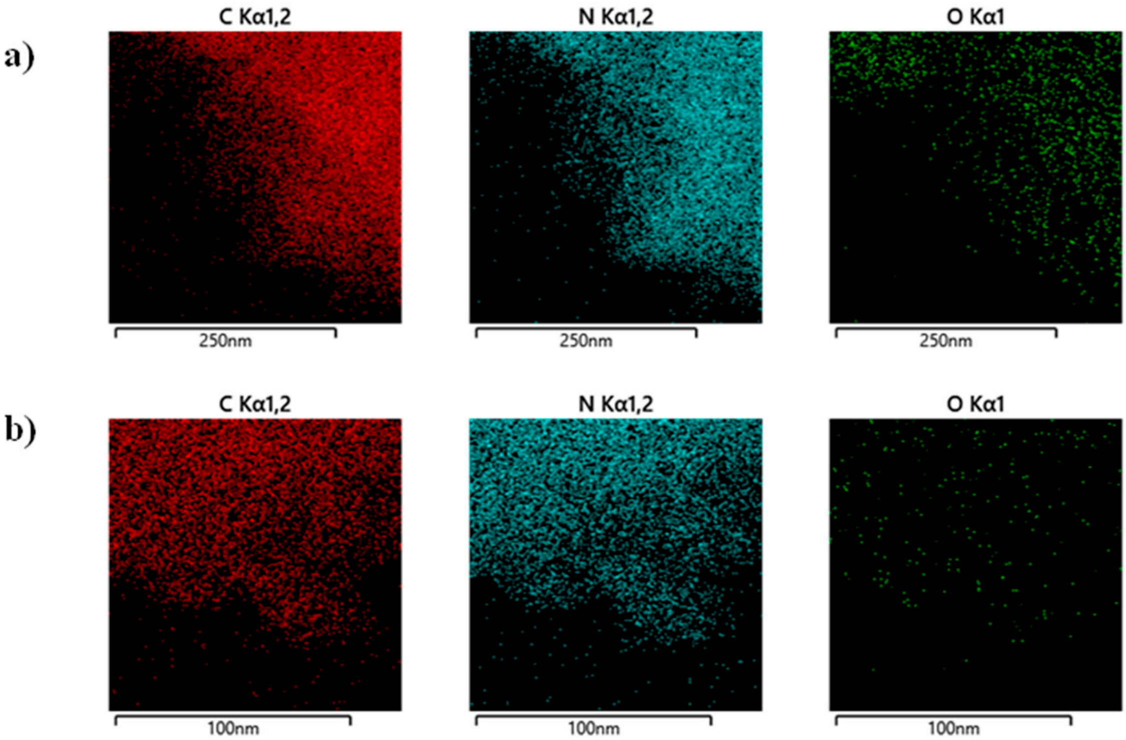

Figure S2. (a) EDS mapping of acid exfoliated CNNF, (b) EDS mapping of ethanol exfoliated.

Table S1. Map sum spectrum of acid exfoliated and ethanol exfoliated CNNF.

Acid exfoliated CNNF							
Element	Line Type	K Factor	K Factor type	Absorption Correction	Wt%	Wt% Sigma	Atomic %
C	K Series	1.339	Theoretical	1.00	40.37	0.71	44.13
N	K Series	1.688	Theoretical	1.00	59.50	0.72	55.76
O	K Series	0.996	Theoretical	1.00	0.13	0.17	0.11
Total					100.00		100.00
Ethanol Exfoliated CNNF							
Element	Line Type	K Factor	K Factor type	Absorption Correction	Wt%	Wt% Sigma	Atomic %
C	K Series	1.339	Theoretical	1.00	52.86	0.53	56.78
N	K Series	1.688	Theoretical	1.00	45.41	0.54	41.82
O	K Series	0.996	Theoretical	1.00	1.73	0.16	1.40
Total					100.00		100.00

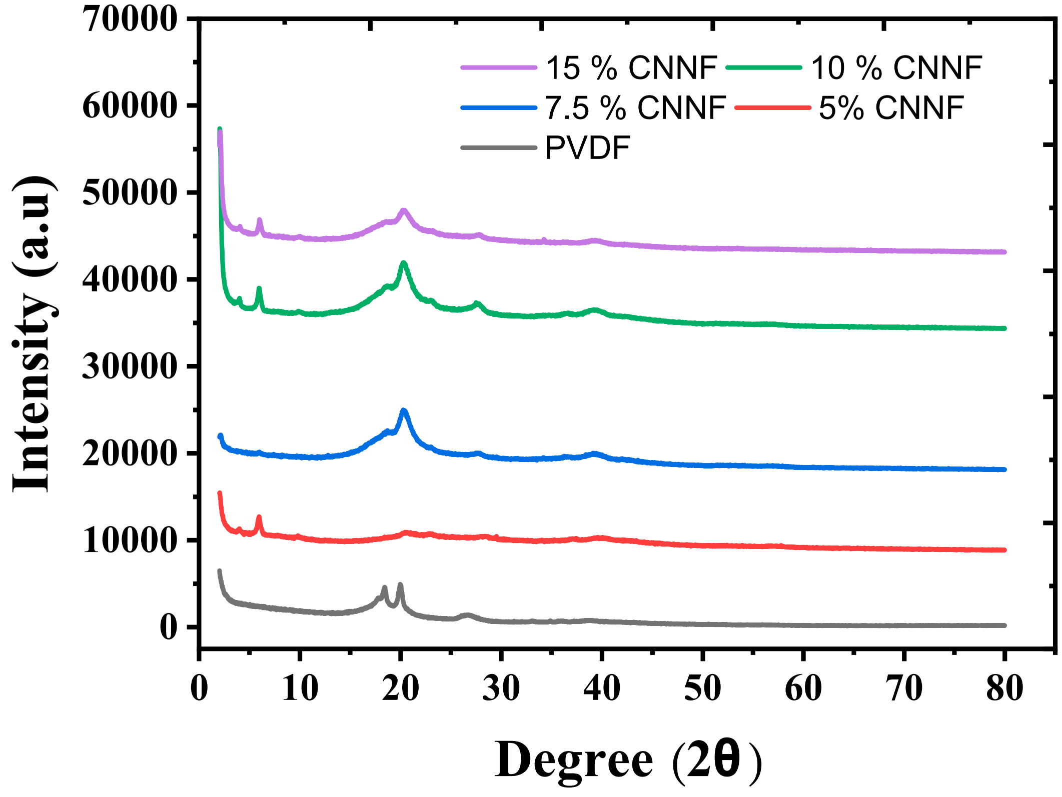

Figure S3. XRD of pure PVDF and film prepared with different concentration of CNNF.

The XRD spectra of prepared film was analyzed, and the β-phase of film was calculated, the β-phase was calculated using following formula

$$\frac{I\beta}{I\beta + I\alpha} \times 100$$

Where Iβ is intensity of β-phase and Iα is intensity of α-phase of PVDF. The β-phase was calculated using this formula where we observed that as the CNNF concertation increased the β-phase also increased, till 7.5% of CNNF, after which the β-Phase was seen to be decreased. Pure PVDF provide 51% after which the β-phase increased to 61% at 7.5% of CNNF after which it decreased to 48% at CNNF15%.
